# Detection of *Taenia saginata* infection mimicking Crohn's disease using video capsule endoscopy

**DOI:** 10.1002/ccr3.1415

**Published:** 2018-03-04

**Authors:** Elchanan Nussinson, Shira Yair‐Sabag, Fahmi Shibli

**Affiliations:** ^1^ Institute of Gastroenterology Emek Medical Center Afula Israel

**Keywords:** Crohn's disease, *Taenia saginata*, *Taenia solium*, tapeworm, video capsule endoscopy

## Abstract

Capsule endoscopy was used to detect intestinal *Taenia saginata* infection in a 19‐year‐old man. The patient was initially believed to have Crohn's disease due to a notable family history of the disease. Capsule endoscopy is a valuable tool for diagnosing tapeworm infection even when Crohn's disease is suspected.

## Introduction

With the increasing incidence of inflammatory bowel disease (IBD), less attention might be paid to intestinal helminth infection in developed countries. Various helminth infections might present with clinical symptoms and radiological findings mimicking Crohn's disease. Infection by the tapeworm *Taenia saginata* is relatively rare in developed countries but is quite common in developing countries, where sanitary conditions are poor and the income is low^1^. Currently, stool microscopy is the gold standard examination for detecting helminth infection. However, it is limited in its ability to identify *Taenia saginata* and has a low sensitivity in detecting mature *Taenia saginata* proglottids and eggs. Coproantigen ELISA techniques and stool PCR are not routinely available in some countries for diagnosing intestinal helminthic infections. As in our case, video capsule endoscopy, which is used occasionally to asses and diagnose Crohn's disease, might also be suitable for detecting various intestinal tapeworm infections, including *Taenia saginata* proglottids and their characteristic structure of uterine branches and genital pores.

## Patient Description

A 19‐year‐old man was admitted to our clinic after experiencing chronic diarrhea that was present since 12 years of age. Upon physical examination, he appeared thin, with a nontender abdomen, and without organomegaly or palpable abdominal masses. His family history included two uncles and cousins with Crohn's disease. Laboratory examination indicated that complete blood count, serum albumin level, liver function test results, serum amylase level, folic acid level, and T4 level were within normal limits. Serum vitamin B12 levels were at the lower limit of the normal range (197 pmol/L), and serum C‐reactive protein (CRP) levels were found to be mildly elevated (7.8 mg/dL, *N* = 0–5 mg/dL) during a previous hospitalization, but was otherwise normal. Stool calprotectin was within normal limits. Microscopic stool examination and stool culture were negative for parasitic ova or bacteria indicative of infection.

**Figure 1:**
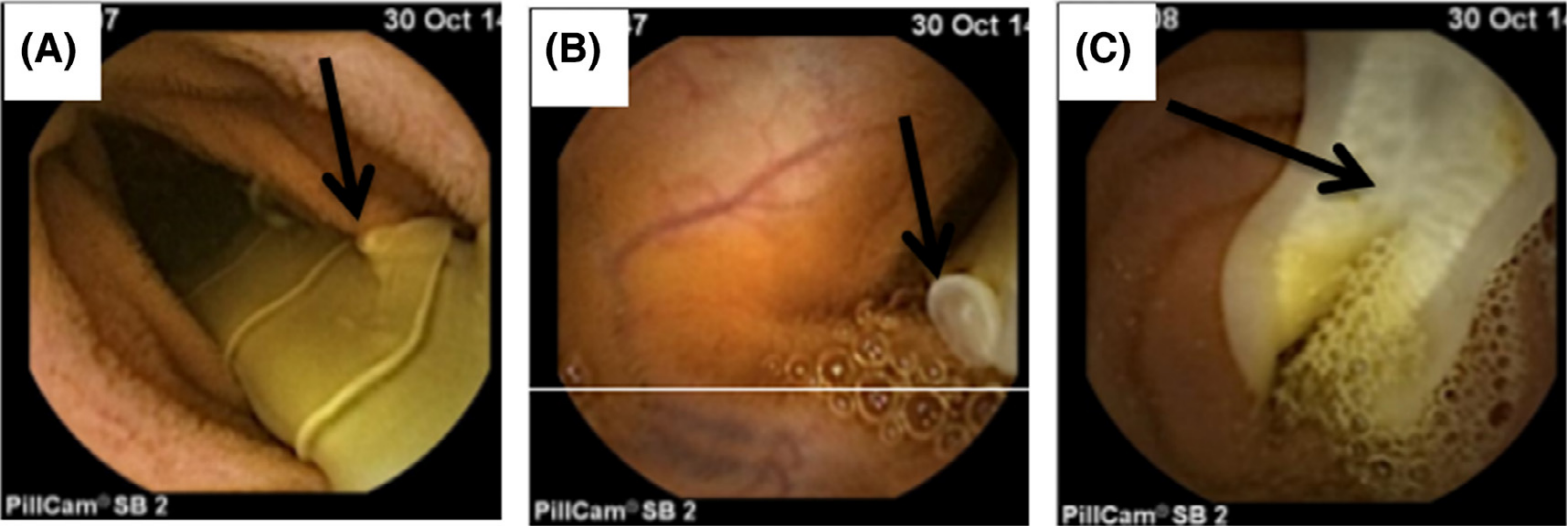
Capsule endoscopy findings. (A) *Taenia saginata* in the small intestine with a genital pore (arrow). (B) A genital pore (arrow). (C) A gravid proglottid with its uterine system (arrow).

The findings of upper gastrointestinal endoscopy and colonoscopy with terminal ileum biopsy were normal. Abdominal computed tomography (CT) showed terminal ileal wall thickening and some filling defects of the small intestine, which were suggestive of Crohn's disease or intestinal parasites. Video capsule examination revealed the presence of *Taenia saginata* strobila with characteristic proglottids and uterine branches and pores that were infesting the small intestine (Fig. 1).

The patient was treated with a single dose praziquantel 10 mg/kg, resulting in complete resolution of his clinical symptoms of abdominal pain and diarrhea. He started to gain weight and reported that the tapeworm was expelled in his stool.

## Discussion

In the last few years, several cases of tapeworm infection (*Taenia* and *Diphyllobothrium latum*) of the small intestine diagnosed using capsule endoscopy have been reported 2, 3, 4, 5, 6, 7. Other intestinal helminthic infections including hookworm (*Necator americanus* and *Ancylostoma duodenal*), whipworm (Trichuris trichiura), and roundworm (*Ascaris*) infections have been diagnosed using capsule endoscopy 8, 9, 10, 11, 12. Additionally, previous reports have described the identification of hookworms (*Necator americanus* and *Ancylostoma duodenale*) using upper gastrointestinal endoscopy and the diagnosis of whipworm (*Trichuris*) infections of the cecum and ascending colon using colonoscopy 10, 12. Some intestinal helminthic infections might produce clinical symptoms and radiological findings that are similar to those of inflammatory bowel disease (IBD) 13, 14, 15. Interestingly, asymptomatic *Taenia saginata* infection in a patient with Crohn's disease being treated with an anti‐TNFa therapy was recently described 16. In our patient with intestinal taeniasis, a family history of Crohn's disease, elevated serum CRP level, and a thickened ileal wall upon abdominal CT prompted us to consider a diagnosis of Crohn's disease. However, upper gastrointestinal endoscopy and colonoscopy did not support this diagnosis. Stool microscopy was negative for parasites, and this approach might be considered insensitive for the detection of mature *Taenia* proglottids and its eggs given the unequal distribution of the eggs in the feces and the destruction of gravid proglottids 17, 18. The technique used in our laboratory examinations for the detection of *Taenia saginata* eggs in the feces is direct microscopy and with prior stool concentration with formalin‐ether. It has a 38% sensitivity (range: 3.9–52.5%) in detection of *Taenia saginata* 19. Another technique, ELISA detection of fecal Taenia solium coproantigens, a secretory antigen produced by the genital system (developed by Allain's group), is reliable, important, and may detect *Taenia saginata* 2.5 times more than microscopy 20 independently on proglottid fecal expulsion or eggs shedding. This technique is especially good for the detection of taeniasis treatment failure as well as taeniasis in the prepatent stage 20.

However, this method is not commercially available, moreover, it has cross‐reaction with other parasites including Ascaris and Trichuris 19 and its specificity in discrimination of *Taenia saginata* from *Taenia solium* is unclear 21, 22.

In our case, only capsule endoscopy revealed small intestinal taeniasis with gravid proglottids and uterine branches and genital pores (Fig. 1). Moreover, capsule endoscopy may be able to identify jejunal ulcers adjacent to *Taenia* parasites in cases with iron deficiency anemia 4, 5. Capsule endoscopy might also help in differentiating between various tapeworms (*Taenia saginata* and *Diphyllobothrium latum*) according to the shape of the uterine branches of their proglottids (i.e., *Taenia saginata* exhibits approximately 15–20 uterine branches of the proglottids, while the uterine branches of *Diphyllobothrium latum* take the form of a central rosette 3, 6).

Radiology studies might help identify helminthic infection of the intestine by demonstrating thin longitudinal filling defects of the intestine 3. However, as in our case, abdominal CT and a small bowel series might indicate a thickening of the jejunum and ileum, which might result in confusion of the diagnosis with IBD 13. Clinically, patients infested with *Taenia saginata* are either asymptomatic or may have nonspecific symptoms, including abdominal pain, diarrhea, nausea, vomiting, weight loss, and iron deficiency anemia. The mature form of *Taenia saginata* might lead to more serious and rare complications, such as obstruction of the appendix, pancreatic duct, or common bile duct 23, 24.

Our patient did not consume pork; thus, infection with *Taenia saginata*, the beef tapeworm, was the most likely diagnosis. However, it is important, albeit difficult, to differentiate between intestinal *Taenia saginata* infection and *Taenia solium* infection (the pork tapeworm infection) given the risk of neurocysticercosis in patients infected with *Taenia solium* 3. The various *Taenia* species can be identified using PCR amplification of the 5.8S ribosomal RNA gene and subsequent restriction enzyme analysis of the PCR product 3, 17, 18 and by multiplex PCR (polymerase chain reaction), targeting the mitochondrial gene; however, their sensitivity might be lower than that of fecal coproantigen ELISA test and is limited by the fecal egg number 25. Furthermore, few laboratories have this molecular tool which is relatively expensive 22. Microscopic counting of the uterine branches of the proglottids that are expelled in stools (more than 12 for *Taenia saginata* vs. 5–10 for *Taenia solium*) and microscopic identification of the scolex shape 3 and the distinct vaginal pore sphincter of *Taenia saginata* 26 can allow for the differentiation between these two species of *Taenia*. Future advanced capsule endoscopy techniques 12 might enable the improved identification of the scolex and the proglottid uterine branches, thereby facilitating endoscopic differentiation between *Taenia saginata* and *Taenia solium*.

## Conclusion

Intestinal helminths infection, including tapeworm infection, should be considered in the differential diagnosis of IBD, even in developed countries. Given the low sensitivity and low negative predictive value of microscopic stool examination for taeniasis and the lack of availability of serological and molecular studies, such as serum and fecal ELISA for parasitic coproantigen antibodies and stool PCR assays (targeting mitochondrial and ribosomal RNA genes), capsule endoscopy may be an additional tool helpful in diagnosing intestinal *Taenia* infection.

## Authorship

EN: drafted and wrote the manuscript, performed the review of the literature, and contributed to the date interpretation. SYS: wrote and drafted the manuscript and the data collection. FS: drafted the manuscript, contributed to data interpretation, and provided patient care and follow‐up. All authors approved the final manuscript as submitted.

## Conflict of Interest

None of the authors have a conflict of interest with any institution or organization.
